# The child’s perception on monitoring inflammatory bowel disease activity

**DOI:** 10.1007/s00431-021-04315-5

**Published:** 2021-11-10

**Authors:** Elsa A. van Wassenaer, Renée R. van der Klift, Mira S. Staphorst, Johanna H. van der Lee, Marc A. Benninga, Bart G. P. Koot

**Affiliations:** 1 Pediatric Gastroenterology, Emma Children’s Hospital, Amsterdam UMC, University of Amsterdam, Amsterdam, The Netherlands; 2 Amsterdam Reproduction and Development, Amsterdam Gastroenterology, Endocrinology and Metabolism, Amsterdam UMC, University of Amsterdam, Meibergdreef 9, Amsterdam, 1105 AZ The Netherlands; 3 Emma Children’s Hospital, Amsterdam UMC, University of Amsterdam, Amsterdam, The Netherlands; 4 Pediatric Clinical Research Office, Emma Children’s Hospital, Amsterdam UMC, University of Amsterdam, Amsterdam, The Netherlands; 5 Knowledge Institute of the Dutch Association of Medical Specialists, Utrecht, The Netherlands

**Keywords:** Patient reported discomfort, Pediatric inflammatory bowel disease, Disease monitoring, Diagnostic testing, Patient preferences

## Abstract

To determine the perception of children with inflammatory bowel disease (IBD) regarding monitoring tests, we first compared the reported discomfort and patient perspective during gastro-intestinal (GI)-endoscopy, magnetic resonance entrography (MRE), and ultrasound (US) and, in a second comparison, patient preference on non-invasive tests (venipuncture, sampling stool and US). A cross-sectional study in children 8–18 years undergoing an US, MRE, and GI-endoscopy for diagnosis or follow-up of IBD. After each procedure, the children filled out the Discomfort during research procedures questionnaire (DISCO-RC). Items of the DISCO-RC are as follows: nervousness, annoyance, pain, fright, boredom, and tiredness. Answers range from “not” (= 0 points) to “extremely” (= 4 points) (range total score: 0–24). Differences between the procedures were assessed with Friedman test, with subsequent Wilcoxon signed-rank test. The children were also asked which non-invasive test they preferred not to undergo regularly (venipuncture, stool-sampling, or US). Answers were analyzed with *χ*^2^-test. Forty-nine patients (27 (55%) female, median age 15 (range 9–17)) were included. The children reported to be most nervous, frightened, and tired after GI-endoscopy (median: 1, 1, 2 points, respectively), equally annoyed by MRE and GI-endoscopy (median 1 point), and equally bored by MRE and US. GI-endoscopy was ranked as most discomfortable, followed by MRE and US (total DISCO-RC scores: 7 vs. 5 vs. 2, *p* < 0.001). Most of the children preferred not to sample stool or perform venipuncture regularly (*n* = 20 (41%, both) (*p* < 0.001)).

*Conclusion*: Our results suggest that the children with IBD report low discomfort after US, MRE, and GI-endoscopy. US is preferred as a monitoring tool, also among non-invasive monitoring tests. GI-endoscopy was most discomfortable.

**Table:** 

**What is Known:** *• Children with inflammatory bowel disease need to be monitored frequently for disease activity.* *• Adult studies — including a systematic review — on acceptability of monitoring tools among IBD patients showed mixed results.*	
**What is New:** *• Children in our study ranked gastro-intestinal endoscopy as most discomfortable, followed by MRE and US.* *• With regard to non-invasive monitoring, most children preferred not to sample stool or perform venipuncture regularly, and preferred US.*

**What is Known:**

*• Children with inflammatory bowel disease need to be monitored frequently for disease activity.*

*• Adult studies — including a systematic review — on acceptability of monitoring tools among IBD patients showed mixed results.*

**What is New:**

*• Children in our study ranked gastro-intestinal endoscopy as most discomfortable, followed by MRE and US.*

*• With regard to non-invasive monitoring, most children preferred not to sample stool or perform venipuncture regularly, and preferred US.*

## Introduction

Children with inflammatory bowel disease (IBD) — a chronic relapsing and remitting condition — need to undergo repeated testing to monitor disease activity [1, 2]. Intensive monitoring is propagated in the current IBD guidelines — also in the absence of symptoms — as treatment of subclinical inflammation has been shown to be related to a better prognosis [3]. This “treating to a target” strategy should, however, be balanced with the patients’ discomfort caused by intensive use of monitoring tools. To balance these competing interests, knowledge into the child’s perception on the different monitoring tools for IBD is needed.

Gold standard diagnostic and monitoring tools in IBD are gastro-intestinal (GI) endoscopies and magnetic resonance enterography (MRE). Frequently used non-invasive monitoring tools are inflammatory markers in blood (e.g., C-reactive protein) and in stool (e.g., fecal calprotectin) [1]. In recent years, intestinal ultrasound (US) is used increasingly as an additional diagnostic and monitoring tool in IBD, providing non-invasive intestinal imaging. Adult studies — including a systematic review — on acceptability of different types of monitoring tools among IBD patients showed mixed results as in some endoscopy, while in others venipuncture had the lowest acceptability [4–6]. The only published pediatric study is a survey among parents of children with IBD in which endoscopy was rated as least comfortable and most worrisome compared to other imaging tests including MRE and US [7]. However, these studies are all online survey studies with a risk for recall and selection bias, and none investigated the child’s perception. To determine the perception of children with IBD regarding the monitoring tools for IBD, we performed a cross-sectional study, first, comparing the discomfort during the gold standard and imaging tests (GI-endoscopy, MRE, and US) and second, evaluating children’s preference for one of the non-invasive tests (venipuncture, sampling stool and US).

## Methods

### Participants

This study is part of a larger study which aims to assess the diagnostic accuracy of bowel ultrasound using ileo-colonoscopy and MRE as the reference standard, in children undergoing an MRE and/or GI-endoscopy for diagnosis or follow-up of IBD. Consecutive children from the Amsterdam University Medical Center, the Netherlands, for whom a GI-endoscopy and/or MRE was requested by their treating physician were asked to participate between August 2019 and May 2021. Inclusion criteria for this sub-study were as follows: 8–18 years of age, sufficient knowledge of the Dutch language to understand the questionnaire and undergoing both a GI-endoscopy, and an MRE for diagnosis or follow-up of IBD. The patients who were unable to understand or fill out the questionnaire or who did not undergo all three investigations were excluded.

### Study procedures

As part of either diagnostic work-up or monitoring, all the children underwent a venipuncture and handed in a stool sample in the month prior to the GI-endoscopy. Before the GI-endoscopy, the patients underwent a polyethylene glycol-based bowel preparation (Kleanprep®) either in the hospital or at home, according to local protocol. The children who were unable to drink the bowel-cleansing fluid themselves were given a naso-gastric tube. All endoscopies were performed under general anesthesia. Within a week of the GI-endoscopy, the children underwent a US examination as well. The children did not take any specific bowel preparation for the US, but they were asked to not take any solid food or gassed fluids 4 h prior to the US. The gel used was warmed prior to the examination. A subset of children (*n* = 25 (51%)) underwent a second US-examination directly after the first, which was used for inter-observer analyses. The MRE was planned based on availability, mostly within a month of the GI-endoscopy. The children were asked to keep nill by mouth for 4 h, and they had to drink 500 cm^3^ contrast fluid (mannitol) prior to the examination. In addition, the children received an intra-venous contrast agent. After the GI-endoscopy, US and MRE, respectively, the children were asked to fill out the ‘Discomfort during research procedures’ questionnaire (DISCO-RC), which is explained in detail below. In case of the GI-endoscopy, the answer evaluated the whole procedure, i.e., bowel preparation and endoscopy. For the MRE, this was not explicitly mentioned. The questionnaires were filled out on the same day as the respective procedures. After the last procedure, the children were asked which of the three procedures they were most willing to undergo a second time, and which of the three procedures they would not want to undergo a second time.

Within a month of the non-invasive tests, the children were also asked which non-invasive test they would mind least to undergo regularly and which non-invasive test they would mind most to undergo regularly. The answering options included: venipuncture, sampling stool, and US.

### DISCO-RC questionnaire

The DISCO-RC consists of six multiple choice questions, all reflecting different forms of discomfort [8]. The questions address nervousness, annoyance, pain, fright, boredom, and tiredness. Each is scored using a 5-point Likert scale and answers range from “not discomforting” (= 0 points) to “extremely discomforting” (= 4 points); hence, the total score ranges from 0 to 24 points. The seventh question of the DISCO-RC is an open-ended question in which the children can give suggestions to reduce discomfort. The questionnaire was developed in Dutch, in children aged 6–18 years, undergoing research procedures, and has been translated into English [9].

### Statistical analyses

Normally distributed values were displayed as means and standard deviations (SD) and non-normally distributed values were displayed as medians with interquartile ranges (IQR). Proportions were displayed as percentages with 95% confidence interval (CI). The difference in discomfort between the US, GI-endoscopy, and MRE was assessed with the Friedman test for non-parametric paired data for all different forms of discomfort separately and for the total DISCO-RC score. In case a significant difference was found, subsequent Wilcoxon signed-rank test was performed, corrected for multiple testing using the Bonferroni method. Corrected *p*-values were displayed. For patients who were excluded because they did not undergo all three investigations, we assessed the total DISCO score as well. Differences in proportions were tested with the chi-squared test. *P*-values < 0.05 were considered significant. Analyses were performed with SPSS v.26.

## Results

A total of 122 patients were asked to participate and of these, 49 patients (27 (55%) female, with a median age of 15 years (range 9–17)) were included in this study. Reasons for exclusion were: not undergoing all three investigations (*n* = 70) and insufficient knowledge of the Dutch language to understand the questionnaire (*n* = 3). Most patients (*n* = 41 (84%)) underwent the diagnostic procedures because they were suspected of IBD. Forty-seven out of 49 (96%) were diagnosed with IBD (36 Crohn’s disease, 5 ulcerative colitis, 6 IBD-unclassified). In the other two, the suspicion was not confirmed. Of the IBD patients, 20% had clinically inactive disease, 36% had mild clinical disease activity, 39% had moderate clinical disease activity, and 5% had severe clinical disease activity, based on the pediatric Crohn’s disease/ulcerative colitis activity index.

### Endoscopy vs. MRE vs. US

**Fig. 1:**
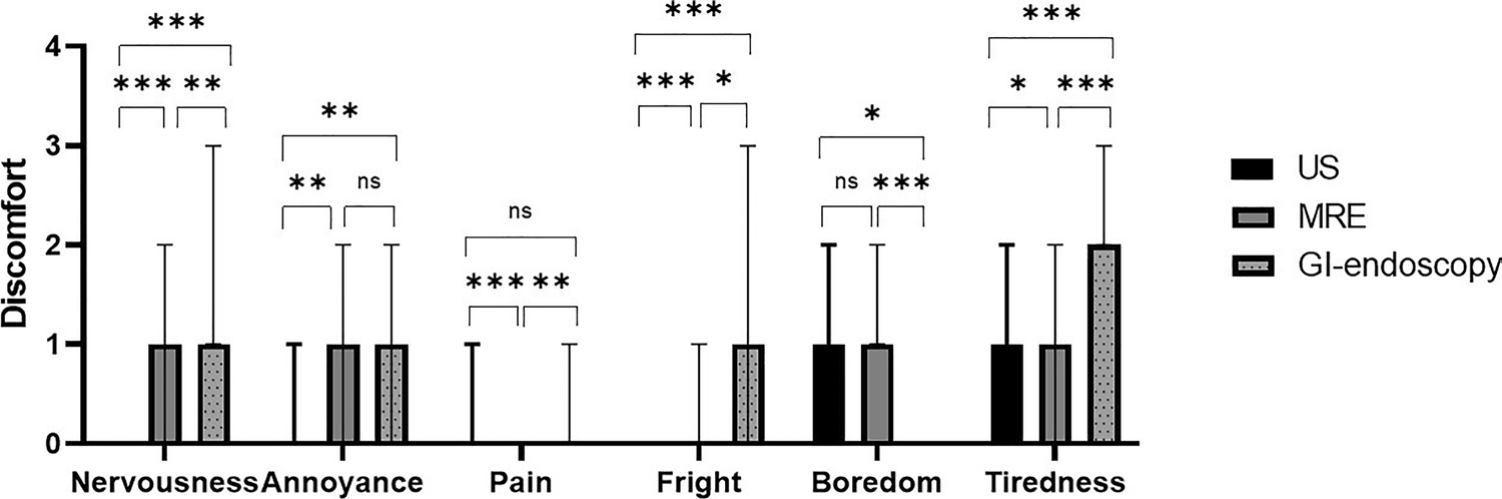
Median scores of all different items of the Discomfort during research procedures questionnaire after the ultrasound (US), magnetic resonance enterography (MRE), and GI-endoscopy, respectively. Whiskers represent interquartile ranges. Corrected *p*-values of analyses are displayed as: *p < 0.05, **p < 0.01, ***p < 0.001

**Fig. 2:**
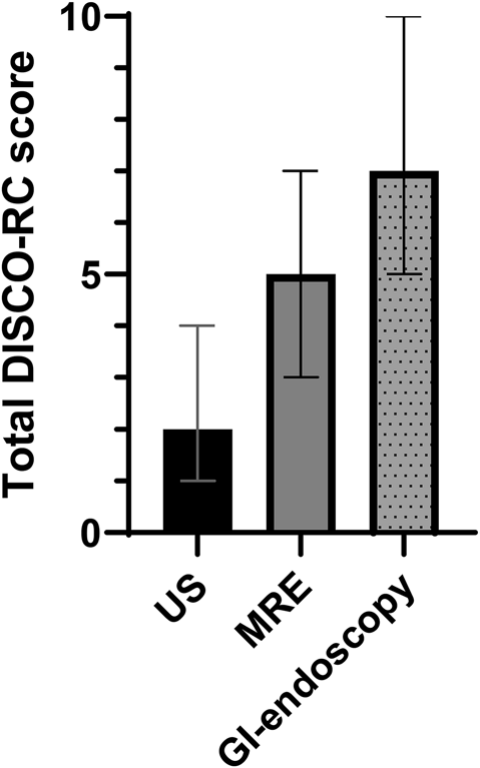
Median total score of the Discomfort during research procedures questionnaire after the ultrasound (US), magnetic resonance enterography (MRE), and GI-endoscopy, respectively. Whiskers represent interquartile ranges. Differences between the total scores were statistically significant (*p* < 0.05)

The answers to the DISCO-RC are displayed in Tables 1, 2, and 3 and Fig. 1. Overall, the reported discomfort was low; the median scores for all questions ranged from 0 (not discomfortable) to 2 (somewhat discomfortable), and the total DISCO-RC score ranged from 2 to 7 on a scale of 0 to 24. The total DISCO score of the included children did not differ significantly from the children who were excluded for not undergoing all three investigations (*p* > 0.05). There was a small but significant difference of 1 point in reported nervousness, fright, and tiredness after GI-endoscopy, compared with the MRE (corrected *p*-values: 0.006, 0.018, < 0.001, respectively), and these items were also significantly higher for MRE compared with the US (corrected *p*-values: < 0.001, < 0.001, 0.021, respectively) (Fig. 1). The children were equally annoyed by the GI-endoscopy and MRE, equally bored by the MRE and US, and the US and GI-endoscopy were ranked as equally painful (corrected *p*-values all > 0.05). In total, the GI-endoscopy was ranked as most discomfortable, followed by the MRE and the US (*p* < 0.001) (Tables 1, 2, and 3; Fig. 2).

**Table 1:** DISCO-RC scores after ultrasound (*n* (%))

**Score subscales**	Not (= 0)	Slightly (= 1)	Somewhat (= 2)	Very (= 3)	Extremely (= 4)	Median score (IQR)
**Nervousness**	41 (84)	6 (12)	0 (0)	2 (4)	0 (0)	0 (0–0)
**Annoyance**	35 (71)	11 (22)	2 (4)	1 (2)	0 (0)	0 (0–1)
**Pain**	32 (65)	15 (31)	2 (4)	0 (0)	0 (0)	0 (0–1)
**Fright**	47 (96)	2 (4)	0 (0)	0 (0)	0 (0)	0 (0–0)
**Boredom**	15 (31)	22 (45)	9 (18)	0 (0)	3 (6)	1 (0–2)
**Tiredness**	28 (57)	17 (35)	3 (6)	0 (0)	1 (2)	1 (0–1)
**Total score**	-	-	-	-	-	**2 (1**–**4)**

**Table 2:** DISCO-RC scores after gastro-intestinal endoscopy (*n* (%))

**Score subscales**	Not (= 0)	Slightly (= 1)	Somewhat (= 2)	Very (= 3)	Extremely (= 4)	Median score (IQR)
**Nervousness**	3 (6)	22 (45)	9 (18)	5 (10)	10 (20)	1 (1–3)
**Annoyance**	23 (47)	11 (22)	5 (10)	4 (8)	6 (12)	1 (0–2)
**Pain**	35 (71)	6 (12)	0 (0)	3 (6)	5 (10)	0 (0–1)
**Fright**	21 (43)	11 (22)	5 (10)	4 (8)	8 (16)	1 (0–3)
**Boredom**	38 (78)	5 (10)	2 (4)	2 (4)	2 (4)	0 (0–0)
**Tiredness**	12 (25)	10 (20)	6 (12)	10 (20)	11 (22)	2 (2–3)
**Total score**	-	-	-	-	-	**7 (5–10)**

**Table 3:** DISCO-RC scores after magnetic resonance enterography (*n* (%))

**Score subscales**	Not (= 0)	Slightly (= 1)	Somewhat (= 2)	Very (= 3)	Extremely (= 4)	Median score (IQR)
**Nervousness**	13 (27)	19 (40)	9 (18)	5 (10)	3 (6)	1 (0–2)
**Annoyance**	13 (27)	23 (47)	4 (8)	7 (14)	2 (4)	1 (0–2)
**Pain**	46 (94)	3 (6)	0 (0)	0 (0)	0 (0)	0 (0–0)
**Fright**	28 (57)	11 (22)	9 (18)	0 (0)	1 (2)	0 (0–1)
**Boredom**	12 (25)	13 (27)	14 (29)	8 (16)	2 (4)	1 (1–2)
**Tiredness**	19 (39)	18 (37)	8 (16)	1 (2)	3 (6)	1 (0–2)
**Total score**	-	-	-	-	-	**5 (3**–**7)**

Most of the children (*n* = 45 (92%, [81–97]%)) reported they would prefer to undergo the US again (*p* < 0.001) and would prefer not to undergo the GI-endoscopy (*n* = 36 (74%, [60–84]%)) or MRE (*n* = 11 (22%, [13–36]%)) again (*p* < 0.001).

### Suggestions to reduce discomfort

The answers to the open-end question of the DISCO-RC are summarized in Table 4. Seven (14%) children gave suggestions to reduce discomfort after the US, 24 (53%) children gave suggestions regarding the GI-endoscopy, and 15 (31%) for the MRE. For the US, suggestions included putting something on the wall to look at (*n* = 1), playing music during the US (*n* = 2), applying less pressure (*n* = 2) or using gel that stings less (*n* = 1). For the GI-endoscopy, most suggestions regarded the taste and volume of the bowel preparation (*n* = 17). Other suggestions included: getting the intravenous access right at first try (n = 2), reducing waiting time (*n* = 2), putting a poster on the wall for distraction or covering a part of the room (*n* = 2). For the MRE, most suggestions regarded the noise during the MRE, for example by playing music (*n* = 12). Other suggestions included not drinking the bowel preparation (*n* = 4) or getting more time to drink it (*n* = 1), receiving more explanation beforehand (*n* = 1), ensuring right room temperature (*n* = 1), and no breath holding (*n* = 1).

**Table 4:** Suggestions to reduce discomfort *n* (%)

	**Ultrasound**	**Gastro-intestinal endoscopy**	**Magnetic resonance enterography**
Suggestions related to time	1 (2)	2 (4)	1 (2)
Suggestions related to distraction	3 (6)	4 (8)	12 (25)
Suggestions related to bowel preparation	-	17 (35)	5 (10)
Suggestions related to the intra-venous puncture	-	2 (4)	1 (2)
Other	3 (6)	1 (2)	1 (2)
No suggestion	42 (86)	23 (47)	29 (59)

### Non-invasive procedures

**Fig. 3:**
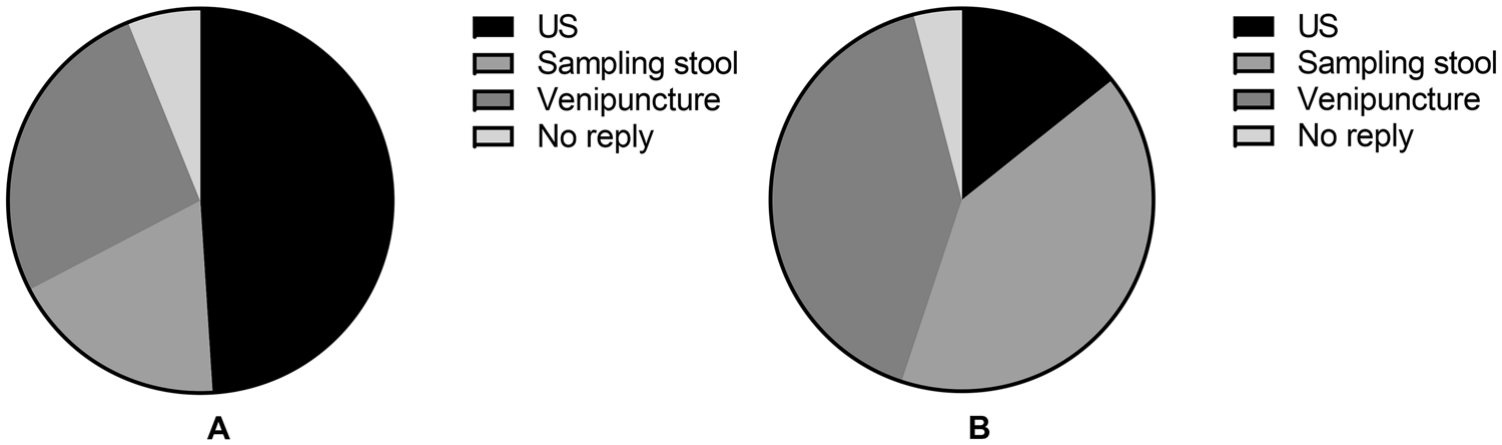
Patient preferences regarding non-invasive procedures. **A**: Answers to “Which test would you mind least to undergo on a regular basis?” **B**: Answers to “Which test would you mind most to undergo on a regular basis?”

The preferences for the non-invasive procedures are displayed in Fig. 3. Most of the children reported they would mind least to undergo a US on a regular basis (*n* = 24 (49%, [36–-63%]), compared to venipuncture (*n* = 13 (27%, [16–40%]) (*p* < 0.001), and compared to sampling stool (*n* = 9 (18%, [10–31%]) (*p* < 0.001). Two children did not have a preference and one child did not answer this question. Most of the children preferred not to sample stool or perform a venipuncture on regular basis (*n* = 20 (41%, [28–55%], both) compared to US (*n* = 7 (14%, [7–27%]) (*p* < 0.001). One child did not have a preference and one child did not answer this question.

## Discussion

This study — comparing discomfort related to US, MRE, and GI-endoscopy in children with IBD — suggests that reported discomfort during these monitoring procedures is relatively low. The children report the lowest level of discomfort after a US and report the least acceptance towards a GI-endoscopy. Also, among the non-invasive monitoring tests, US was preferred above stool sampling and venipuncture in our cohort.

According to IBD guidelines, disease activity should be monitored periodically after diagnosis, to assess treatment response and disease flares [2]. GI-endoscopy and MRE are the gold standard monitoring tools for colon and small bowel respectively, but both are requested with reticence in the pediatric population, among others, because of presumed discomfort as they require i.v. cannulas, bowel preparation, general anesthesia, and/or contrast agents. US does not require bowel preparation or i.v. cannulas and is increasingly applied as non-invasive cross-sectional monitoring tool for both small and large bowel [10]. Adult data suggest good diagnostic accuracy of US compared to GI-endoscopy (area under the receiver operating characteristics curve: 0.94) [11] and compared to MRE (sensitivity and specificity 90% and 96% for US vs. 93% and 93% for MRE) [12]. Our results confirm that patients do, indeed, prefer this non-invasive monitoring tool, although interestingly reported discomfort after GI-endoscopy, and MRE was relatively low as well. Regarding the GI-endoscopy, it seems that discomfort is mainly caused by slight fright and nervousness, possibly prior to the procedure, but perhaps the general anesthesia reduces experienced discomfort after the procedure.

Our results are in line with a previous study in 618 adults with IBD, in which acceptability and perceived utility towards the same diagnostic procedures were measured using a visual analogue scale (VAS) ranging from 1 (low acceptability) to 10 (high acceptability). In this study, both venipuncture and US scored higher (VAS 9.3 [8.8–9.7], and 9.3 [8.7–9.7] respectively), compared with ileo-colonoscopy (VAS 6.7 [4.3–8.9], *p* < 0.001) and MRE (VAS 8.0 IQR [5.0–9.2], *p* < 0.001) [4]. In another survey study in 210 adult IBD patients, the level of comfort and level of understanding of different tests was analyzed using a 6-point Likert scale. In this study, 79% of patients reported a low level of comfort with venipuncture vs. 41% for colonoscopy and 13% for medical imaging (i.e., US and MRE), whereas the level of understanding of the need for the test was highest for the ileo-colonoscopy (87% reported a high level of understanding, compared with 64% and 75% for venipuncture and medical imaging, respectively), suggesting an association between level of comfort and understanding in these patients [5]. However, discomfort caused by diagnostic procedures is a sum of different psychological and physical sensations, and these previous studies did not analyze the different forms of discomfort, but only discomfort/acceptability as one overlapping term. Moreover, perception of discomfort in children may be different than in adults.

The parent perspectives on diagnostic tests for children with IBD have been studied before in a small online survey study (*n* = 28), including mostly mothers (93%), which suggested the colonoscopy to be the least comfortable as well, compared to US, CT, and MRE. However, the small sample size, and risk for recall and selection bias due to its online study design may have limited this study [7]. Moreover, the child’s perspective has not been studied yet. Our study is the first to assess different forms of discomfort in the pediatric IBD population. The results of our study suggest that discomfort during an US is mostly caused by boredom and tiredness, while discomfort during an MRE is also caused by nervousness and annoyance. The GI-endoscopy scored the highest on fright, nervousness, and tiredness. The DISCO-RC has been used before to describe the child’s perspective on research procedures. In a previous study, 77 children aged 10 ± 1.8 years filled out the DISCO-RC after undergoing an US (mostly echocardiograms) and 89 children after undergoing an MRI (mostly of the head) [9]. The children in our study seemed to score slightly lower on the different subsets of discomfort after the US (average sub-score 1.4 vs. 0.0) and the MRE (average sub-score 1.6 vs. 1.0). This might be explained because most (83%) children in the previous study were healthy and underwent the procedures for research purposes only and may have been less accustomed to hospital visits.

With regard to non-invasive monitoring tools, most of the children preferred the US over sampling stool and venipunctures, although this was not assessed with a validated questionnaire. This preference of US over stool sampling is confirmed by the two previously mentioned studies in adults [4, 5]. In the current study, we did not analyze underlying reasons for this preference. However, in adults, embarrassment and dirtiness feeling are often reported [4], and a study in 72 teenagers with IBD suggested that the transfer of the stool to the container with a scoop is the most disturbing part of the sampling procedure [13].

The strengths of this study are the use of a validated questionnaire to measure discomfort in a consecutive patient sample. In addition, the questionnaire was administered on the same day as the procedure by the patients themselves, minimizing recall bias. Lastly, our patient population is a representative sample of the pediatric IBD population, with respect to age, gender, and diagnoses. There are some limitations to this study as well: a subset of patients (51%) in our study underwent two US examinations directly after one another, possibly affecting the reporting on the level of discomfort and especially boredom during US. However, this would mean that US is even perceived more positive than reported in this study. In addition, the DISCO-scores after MRE may have been biased by the fact that the MRE was the last procedure in most cases, and children may have been more familiar with medical procedures by this time. Finally, the comparison between the different non-invasive tools was not done with a validated tool, and these results should thus be interpreted with caution.

In conclusion, our results suggest that pediatric patients with IBD overall report low discomfort after US, MRE, and GI-endoscopy. US is reported as the preferred monitoring tool, also among the non-invasive monitoring tests, and GI-endoscopy was rated as most discomfortable. According to the participants of this study, discomfort can be reduced by providing distraction during the procedures, e.g., by playing music and in case of the GI-endoscopy by improving the taste and volume of the bowel preparation. These findings support the emerging use of US as tool to evaluate disease activity in pediatric IBD. In all, these results can help pediatric gastroenterologists in providing patient-centered care when monitoring children with IBD.

## Data Availability

Data will be made available upon reasonable request.

## Abbreviations

CI: Confidence interval
DISCO-RC: Discomfort during research procedures questionnaire
GI: Gastro-intestinal
IBD: Inflammatory bowel disease
IQR: Interquartile range
MRE: Magnetic resonance enterography
SD: Standard deviation
US: Ultrasound
